# From real world data to real world evidence to improve outcomes in neuro-ophthalmology

**DOI:** 10.1038/s41433-024-03160-8

**Published:** 2024-06-06

**Authors:** Blake D. Colman, Zhuoting Zhu, Ziyi Qi, Anneke van der Walt

**Affiliations:** 1 Department of Neuroscience, Central Clinical School, Faculty of Medicine, Nursing and Health Science, Monash University, Melbourne, VIC Australia; 2 Department of Neurology, Alfred Hospital, Melbourne, VIC Australia; 3 Centre for Eye Research Australia, Ophthalmology, University of Melbourne, Melbourne, VIC Australia; 4 Ophthalmology, Department of Surgery, University of Melbourne, Melbourne, VIC Australia

**Keywords:** Vision disorders, Outcomes research

## Abstract

Real-world data (RWD) can be defined as all data generated during routine clinical care. This includes electronic health records, disease-specific registries, imaging databanks, and data linkage to administrative databases. In the field of neuro-ophthalmology, the intersection of RWD and clinical practice offers unprecedented opportunities to understand and treat rare diseases. However, translating RWD into real-world evidence (RWE) poses several challenges, including data quality, legal and ethical considerations, and sustainability of data sources. This review explores existing RWD sources in neuro-ophthalmology, such as patient registries and electronic health records, and discusses the challenges of data collection and standardisation. We focus on research questions that need to be answered in neuro-ophthalmology and provide an update on RWE generated from various RWD sources. We review and propose solutions to some of the key barriers that can limit translation of a collection of data into impactful clinical evidence. Careful data selection, management, analysis, and interpretation are critical to generate meaningful conclusions.

## Abstract (zh)

真实世界数据(RWD)定义为在常规临床护理期间所产生的所有数据。其中包括电子健康记录、特定疾病登记、影像学数据库以及与管理数据库的数据链接。在神经眼科学领域, RWD和临床实践的交叉为理解和治疗罕见病提供了前所未有的机遇。然而, 将RWD转换为真实世界证据(RWE)提出了一些挑战, 如数据质量、法律和伦理考虑以及数据源的可持续性。本综述探讨了当前神经眼科中现有的RWD来源, 如患者登记和电子健康记录, 并讨论了数据收集和标准化面临的挑战。我们聚焦于神经眼科学需要回答的研究问题, 并提供从各种RWD来源生成的RWE的最新信息。我们对一些可能限制将数据集转化为有影响力的临床证据的关键障碍进行审查并提出解决方案。仔细的数据选择、管理、分析和解释对于得出有意义的结论至关重要。

## Introduction

The United States Food and Drug Administration (FDA) defines real-world data (RWD) as **“**data relating to patient health status and/or the delivery of health care routinely collected from various sources” [1]. Real-world data are generated more rapidly and at much lower cost than data generated in randomised control trials or prospective observational studies. Data sources include electronic health records, disease-specific medical research registries, product registries, and data from wearable and mobile devices. The term ‘big data’ represents the large and complex nature of these data, which can be explored using non-traditional data processing approaches that include various statistical techniques and artificial intelligence (AI). RWD assumes paramount importance when considering the identification and management of rare diseases. Recognised globally as a priority area for healthcare, rare diseases pose unique challenges stemming from a fragmented understanding of their mechanisms and natural history, impeding the development of effective management strategies. Compounded by delayed recognition arising from clinician unfamiliarity, these challenges emphasise the critical need for increased research investment and establishing targeted infrastructure, including data registries, international disease classification systems and biobanks. Collaboration amongst holders of RWD is pivotal in translating such data into real-world evidence (RWE) to address important clinical questions and outcomes in these conditions.

The intersection of RWD and neuro-ophthalmology, therefore, represents a transformative era in medical research, offering unprecedented opportunities to increase the understanding and treatment of these rare diseases (summarised in Table 1). However, this process is challenging, necessitating the establishment of key principles that can serve as a framework for collaborative research. Data quality and harmonisation, legal and ethical considerations to share data across jurisdictions and sustainability of data sources must be considered. As medical registries and data linkage projects in neuro-ophthalmology are relatively early in their development, addressing these concerns is critical to moving the field forward sensibly.

**Table 1:** Neuro-ophthalmological diseases and questions that can be addressed using real-world data.

Epidemiology
- Prevalence and determinants of neuro-ophthalmological diseases in different populations
- Temporal trends in the prevalence of neuro-ophthalmological diseases in different populations
- Demographic trends or variations in the occurrence of these diseases in different settings and countries
Disease Behaviour
- Defining disease symptoms, disease course, phenotype, and prognosis in specific populations
- Predictors of long-term outcomes
- Natural disease course of neuro-ophthalmological diseases
- Impact of comorbidities, health behaviours, genetics and epigenetics, and environment
- Impact of pregnancy, breastfeeding on disease behaviour and outcomes
Diagnostic criteria
- Assess the performance of diagnostic tests and diagnostic criteria in different populations
- Evaluate the time interval between symptom onset, accurate diagnosis and outcome
Disease outcomes
- Study the measurement properties and usefulness of clinical and visual outcome measures
- Evaluate patient reported outcomes as disease outcome measurements in different populations
- Define clinically meaningful improvements by comparing the interrelation between patient rated and clinician-assessed outcome measures
Therapeutic effectiveness of disease therapies and interventions
- Definitions and predictors of treatment response/non-response
- Comparative effectiveness of treatments in different scenarios, for example, surgical interventions in vision-threatening idiopathic intracranial hypertension
- Evaluating treatment strategies, for example, acute interventions in ischaemic optic neuropathies
Quality of care and healthcare utilisation
- Economics and cost–benefit evaluation of treatments and interventions
- Utilisation of diagnostic strategies and treatments in clinical practice
- Benchmark quality of care between health services based on time-to-diagnosis and outcome assessments.
- Identify disparities in healthcare utilisation and outcomes based on demographic and socio-economic factors.
Treatment safety and tolerability of disease therapies
- Safety and risk/benefit assessments, including delayed adverse events, and cumulative risk of adverse events.
- Comparative safety and tolerability between treatments
- Evaluate factors that determine or predict safety and tolerability, including comorbidities, health behaviours, concomitant medications, race and ethnicity, and therapy adherence and persistence.
- Evaluate reproductive safety, including to the mother, unborn foetus and newborn.

This review provides an overview of existing RWD sources in neuro-ophthalmology and discusses challenges to data collection and data standardisation. We provide a conceptual framework to try and resolve some of the barriers facing RWD translation in neuro-ophthalmology into RWE.

### Existing RWD sources in neuro-ophthalmology

We identified sources of RWD by searching PubMed and EMBase using the terms (“neuroophthalmological”[All Fields] OR “neurology”[Mesh]) AND (“big data”[Mesh] OR (“real world data” [Mesh]) OR “clinical research”[Mesh] OR “machine learning”[Mesh] OR “databases as topic”[Mesh]) OR “wearables” [Mesh]” OR “home monitoring” [Mesh].

RWD sources are generated during the routine provision of clinical care, encompassing a broad spectrum of health-related data, service utilisation and interventions received by individuals during care [1, 2]. These sources, often drawn from diverse settings and enriched by data-sharing collaborations, are typically developed for non-research purposes and clinical interpretation of analysed data is considered “secondary use”. This requires meticulous attention to develop research questions and hypotheses to ensure the reliability and validity of any research findings generated. Biases common in RWD arise from variations in data collection, diagnostic coding inaccuracies and selection bias and can confound the interpretation of research findings. A clear analytical approach needs to be in place and will be discussed later in this review.

## Registry data

### Registry real-world data

RWD registries, often observational and prospective in design, provide a systematic framework for the collection and analysis of patient information and clinical data [3]. They allow for the longitudinal assessment of patient populations, treatment responses, and disease trajectories and are typically defined according to the inclusion criteria and the type of data collected. Strategically collected in readily accessible databases, registries represent versatile and valuable resources that can support a range of study designs, identify potential research subjects or conditions, and serve as a foundational resource for future investigations (see Table 1). Table 2 summarises the currently available registries with neuro-ophthalmological data.

**Table 2:** Medical and disease registries in neuro-ophthalmology.

Research Registry	Country of Origin	Provider	Type	Duration	Size.	Sampling methods	Platform	Included population	Information obtained
INSIGHT Health Data Research Hub for Eye Health [8, 11]	United Kingdom	Moorfields Eye Hospital NHS Foundation Trust	Multi-site [2], site-based prospective observational cohort	2019 – present	28+ million eye images and associated clinical metadata	Purposive sampling, National opt-out platform	NHS secure data environment	All patients attending eye clinic at Moorfields Eye Hospital and University Hospital Birmingham.	Pseudonymised and irreversibly deidentified longitudinal demographic, medical and ophthalmological data
Intelligent Research and Sight Registry (IRIS) [7, 9, 10]	United States of America	American Academy of Ophthalmology	Multi-site, clinician specific prospective cohort	2014 – present	72+ million patient records	Clinician entered electronic medical record data	Cloud-based	N/A	Demographic, medical and ophthalmological history, clinical examination findings, results, procedures and medications
Intracranial hypertension registry [4, 14]	United States of America	Oregon Health and Science University	Multi-site (37 countries), disease-specific prospective observational cohort	2003 – present	N/A	Purposive sampling, written consent, referred through physician or self-enrolment	RedCAP	Eligible patients with idiopathic intracranial hypertension	Patient information and neuro-imaging library
The IIH Database: The IIH:LIFE study [12, 13]	United Kingdom	University Hospitals Birmingham (UHB) NHS Foundation Trust	Single site, disease-specific prospective observational cohort	2012 – present	650+ patients	Purposive sampling, written consent	Bespoke database held within NHS secure data environment	Eligible patients with idiopathic intracranial hypertension	Demographic, medical, neuro-ophthalmological history, clinical examination findings and results
University of Illinois at Chicago (UIC) Neuro-Ophthalmology Registry [3]	United States of America	University of Illinois at Chicago	Site-based prospective observational cohort	N/A	N/A	Purposive sampling, written consent, all patients reviewed in neuro-ophthalmology outpatient clinic	RedCAP	N/A	Demographic, medical, neuro-ophthalmological history, clinical examination findings and results
Neuro-Ophthalmology Database (NODE) [6]	Australia	Monash University	Multi-site [2] site-based prospective observational cohort	2019 – present	3500+ patient records	Purposive sampling, opt-out consent, all patients reviewed in neuro-ophthalmology outpatient clinic	RedCAP	Adult patients (>18), all sexes with neuro-ophthalmic symptoms/diagnosis	Demographic, medical, neuro-ophthalmological history, clinical examination findings and results
MSBase and NMOBase [5, 16, 17]	Australia	MSBase Foundation Ltd	Multi-site (43 countries), disease-specific prospective observational cohort	2004 – present	96,000+ patient records	Purposive sampling, written consent, clinician entered electronic medical data	Web-based registry	Eligible patients with MS, Neuromyelitis optica spectrum disorders, MOG-antibody disease. Captures presenting symptoms and long-term outcomes	Demographic, clinical and limited paraclinical information
MGBase [49]	Australia	MSBase Foundation Ltd	Multi-site (6 countries), disease-specific prospective observational cohort	2021 – present	415 patient records	Purposive sampling, written consent, referred through physician or self-enrolment	Web-based registry	Eligible patients with ocular myasthenia gravis	Demographic, clinical and limited paraclinical information
Sight Outcomes Research Collaborative (SOURCE) [20]	United States of America	University of Michigan	Multi-site			Algorithm-led data extraction direct from medical records	Web-based registry	N/A	Demographic, clinical examination, billing codes, diagnoses, therapeutic interventions and radiology
The Vienna Idiopathic Intracranial Hypertension (VIIH) Database [50].	Vienna, Austria	Medical University of Vienna	Single site	2021 – present	113 patient records	N/A	N/A	Eligible patients with idiopathic intracranial hypertension or idiopathic intracranial hypertension without papilloedema	Demographic, medical, neuro-ophthalmological history, clinical examination findings and results
Vision and Eye Health Surveillance System (VEHSS) [23, 24]	United States of America	Centres for Disease Control (CDC)	Multifaceted surveillance dataset with mixed retrospective and prospective collection	2018 – present		Combination of national surveys, examination-based studies, electronic health records, co-existing medical research registries and administrative claims records	Cloud-based	17 specific ocular diseases and 93 clinical sub-classifications	Composite prevalence estimates of visual loss

Registries can be categorised according to the primary objective of the data collection. Patient-based registries collect data from individual patients regardless of their specific medical condition and may encompass diverse patient populations. These registries create extensive datasets on health-care utilisation patterns, treatment effectiveness and longitudinal outcomes across various diseases. Disease-based registries (e.g. Intracranial Hypertension Registry [4], MSBase [5], and NODE [6]) collect data surrounding a specific diagnosis or a group of related conditions. By accumulating information from individuals diagnosed with the targeted condition, disease-based registries facilitate advancements in the understanding of disease epidemiology and treatment outcomes. Surveillance and incidence registries (e.g. The American Academy of Ophthalmology American Academy of Ophthalmology IRIS (Intelligent Research In Sight) Registry) [7] can be used to monitor trends and inform on interventions used to mitigate health risks. Registries can be site-based (IIH:LIFE study) [8], include multiple sites in a single country [7] or internationally [5]. Data collection in these registries is purposeful and generated according to a core dataset at certain time intervals.

### Real world evidence generated from registries

Real-world evidence generated from patient registries is emerging and influencing clinical practice. Although much of this is focused on outcomes in ophthalmological diseases, clinical neuro-ophthalmological questions are also addressed. Work published by the American Academy of Ophthalmology IRIS Registry identified 27,339 eyes with giant cell arteritis (GCA) to evaluate the association between the incidence of GCA and month and season. No relationship was found, contradicting previous, smaller studies [9]. An evaluation of prevalence and associated factors in thyroid eye disease (TED) in 41,211 patients identified in IRIS identified new observations such as an unimodal age distribution (highest prevalence between 50 and 59 years) and racial variations [10]. Several prospective registries for Idiopathic intracranial Hypertension are driving changes in clinical practice and providing evidence-based guidance on management. The longitudinal UK-based IIH: LIFE Study is a single-site cohort with deeply phenotyped data. The study has enhanced our understanding of prognostic factors associated with visual impairment in IIH, such as the severity of papilledema, disease duration and body mass index [11]. The IIH: LIFE cohort describes disease behaviour and outcomes in sub-cohorts of patients with IIH, such as asymptomatic disease [12]. It also evaluated outcomes in pregnant women with IIH and found little to suggest an association between adverse outcomes and pregnancy [13]. The USA-based IIH Research Foundation’s IH Registry evaluated the use of acetazolamide in 158 pregnancies in women with IIH. Acetazolamide exposure occurred in 50 pregnancies before week 13 of gestation, and no adverse effects in any exposed pregnancy were noted [14]. The Swedish IIH study provided further insights into the incidence and contributory factors to this disease using a national population-based design and capturing 902 individuals with disease. Strong associations with both arterial hypertension (OR 17.5), renal failure (OR 13) were shown in patients with IIH when compared to controls, while further evidence for systemic lupus erythematosus (OR 13.8) as an independent risk factor for IIH was also demonstrated [15]. Real-world outcomes in treated versus untreated patients with acute optic neuritis associated with multiple sclerosis were described in 1317 patients from the international MSBase registry. This study confirmed that acute treatment improved visual outcomes and reduced the risk of progression to MS at a median follow-up time of 5.2 years (IQR 2.4–9.3) [16]. A RWE Study of the outcomes in 206 AQP4-IgG+ patients from the MSBase/NMOBase registry demonstrated that a younger age, exposure to azathioprine (HR = 0.46, *p* < 0.001) and mycophenolate mofetil (HR = 0.09, *p* = 0.012) are all associated with a reduced risk of relapse [17]. A slower increase in EDSS was associated with disease-modifying treatment exposure to azathioprine, mycophenolate mofetil and rituximab. Registries can also be used to develop quality improvement in services. The Australian multi-site Neuro-ophthalmology Database (NODE) Registry [6] established a consensus agreement on triage categories for neuro-ophthalmological conditions by applying a modified Delphi approach to the assignation of neuro-ophthalmological conditions such as IIH, cranial nerve deficits, double vision, headache with visual symptoms and papilledema.

### Registry challenges

Data quality refers to the overall attributes and properties of the dataset that determine the adequacy for fulfilling the purposes of its intended usage [18]. The quality of data in rare disease registries may be compromised by several factors, including the low prevalence of disease which can limit statistical power and reliability of the data, and variability in data collection influencing the accuracy of the information gathered. The heterogeneity of neuro-ophthalmological conditions can also be challenging, and condition-specific registries, such as IIH:Life, often yield better targeted results. Data entry and maintenance are labour-intensive and often require clinicians to enter data in real time. This can necessitate limitations on minimum and core datasets. Nevertheless, the challenges of managing such registries have significantly reduced through increased computing power, enhanced storage facilities, improved accessibility and data security. Such advancements vastly expand the ease with which ‘big data’ can be obtained, allowing the construction of detailed and inclusive registries that amplify clinical care by establishing large observational cohorts and reducing the inherent selection bias that may arise with more limited disease-specific databases.

## Data extraction from electronic health records and administrative databases

### Electronic health records and administrative databases

Electronic health record (EHR) sets constitute expansive repositories of comprehensive patient-focused data created from routine clinical encounters. These digital records are maintained by healthcare providers and organisations such as hospitals, clinics, and individual physician practices and offer a centralised and accessible platform for healthcare professionals to review health information, make informed clinical decisions, coordinate care among providers and track health outcomes over time. Similarly, administrative databases, including insurance claim and billing records, provide further insight into healthcare utilisation, service delivery and reimbursement patterns. Leveraging these rich sources of RWD allows for further large-scale population-based studies and can empower evidence-based decision-making in healthcare policy and practice.

### Real-world evidence from electronic health records and administrative databases

The Sight Outcomes Research Collaborative (SOURCE) [19], originating from the University of Michigan, is a large repository of ophthalmological data extracted directly from the electronic medical records (Epic Systems) of numerous ophthalmology centres across the USA. It uses a purpose-designed data extraction code and ensures deidentification by removing protected health information. SOURCE seeks to aggregate data on patient demographics, diagnoses identified from ICD (International Classification of Diseases) billing codes, clinical examination findings and therapeutic intervention. Application of the extraction algorithm was demonstrated to have a far higher positive and negative predictive value for detecting a specific ophthalmological disease than conventional billing code-based approaches alone, highlighting the value of direct EHR data capture in creating more robust datasets [20]. Though limited published outcomes using SOURCE data exist, a recent multi-centre study utilised EHR data on 36,548 individuals from six ophthalmological centres and found a reasonable predictive accuracy in determining those with glaucoma progression requiring surgery [21]. Identifying cases using ICD codes could be amplified by adding additional criteria, such as magneticresonance imaging Results (MRI). Acute optic neuritis can, for example, be better identified If ICD codes are combined with a positive MRI finding [22]. Algorithms may, therefore, need to be adjusted depending on the condition of interest.

The Vision and Eye Health Surveillance System (VEHSS) [23] provides a more comprehensive platform by aggregating data from multiple sources. Initiated by the Centres for Disease Control (CDC) Vision Health Initiative and the non-partisan and objective research organisation (NORC) at the University of Chicago, this surveillance registry was created to obtain a greater estimate of eye health trends, the prevalence of ocular disease and the utilisation of eye care services across different demographic groups and geographic regions in the USA. By leveraging data extraction techniques and ensuring deidentification, VEHSS obtains information directly from electronic medical records, insurance claims databases, population surveys and a range of public health agencies and registries. This includes sources such as Medicare and Medicaid, as well as data obtained from the American Academy of Ophthalmology’s IRIS registry. While expansive, it should be recognised that limitations can arise, including issues surrounding inconsistencies in datasets and reliance on self-reported data, which may affect the accuracy of prevalence estimates. One key example involves the real-world application of VEHSS data in obtaining an updated prevalence of visual loss and blindness within the United States. While this found rates to be 68.7% higher than previous estimates [24] such a significant disparity questions the accuracy of this finding and underscores the inherent challenges and limitations when relying on non-standardised data.

### Electronic health records and administrative database challenges

Data extracted from health records or other administrative databases are often limited by non-standardised data entry. The construction of datasets depends on investigators designing data extraction tools or algorithms restricted by the number of variables that can be included. Missing data are challenging to assess and can lead to underestimation of a research problem. An example is studies that rely on ICD coding. The data accuracy of ICD codes is threatened by diagnostic errors, variability in individual coding assignments or local protocols, and various administrative factors relating to the direct entry of information [25]. A previous systematic review by Hamedani et al. [26] highlighted these difficulties, identifying a marked variability in diagnostic accuracy for a range of common neuro-ophthalmic conditions including idiopathic intracranial hypertension, giant cell arteritis, optic neuritis, neuromyelitis optica and myasthenia gravis. Nevertheless, this data source holds considerable promise due to global use and nomenclature commonality, allowing for comparability in the systematic recording and reporting of health data and outcomes between hospitals, regions and countries. The integration of standardised terminologies like SNOMED-CT (Systemised Nomenclature of Medicine Clinical Terms) aims to address these challenges by providing a common granular vocabulary for recording clinical concepts [27]. By leveraging elements like SNOMED-CT, researchers can design more accurate data extraction algorithms and tools, incorporating sensitivity analyses to mitigate biases and errors arising from missing data.

## Data linkage to government, medication prescribing information and other administrative databases

Tapping into existing healthcare data can uncover valuable health insights, especially when these data sources are linked. Associations between the eye and systemic disease [28, 29] have been explored in artificial intelligence (AI) projects (particularly using a deep learning approach) that offer a revolutionary approach to understanding these relationships [30–32]. AI models can identify patterns in the retina or optic nerve associated with systemic diseases not recognisable to humans. However, the lack of extensive datasets linking ophthalmic data to systemic disease information limits the ongoing development and validation of such AI models. Leveraging routinely collected data is an attractive option for achieving the necessary scale of data for AI in this field.

Currently, data linkage projects in ophthalmology are still in their early stages and are not focused on neuro-ophthalmological disease outcomes. Notable projects that link retinal imaging and systemic diseases include the Alzeye initiative led by the University College London in the United Kingdom [33]. The AlzEye linked high-resolution retinal imaging data, including retinal photographs and OCT scans from patients aged 40 years and older attending Moorfield’s Eye Hospitals with Hospital Episode Statistics (HES), which is a national database comprising of all hospital and emergency admissions and outpatient appointments in England. It has employed a privacy-by-designed third-party linkage approach to link over 2 million retinal photographs and OCT scans of over 250,000 individuals to the HES database. Recent work, using 84,753 high-quality OCT images from 53,159 individuals in the UK Biobank, calculated the gap between OCT-predicted age and chronological age, named the OCT age gap. For each 5-year increase in the OCT age gap, there was an 8% increased mortality risk (hazard ratio [HR] = 1.08, CI:1.02–1.13, *P* = 0.004), demonstrating that the OCT age gap can be used as a marker of the risk of mortality [34].

A similar Australian data linkage project led by the Centre for Eye Research Australia (CERA) utilises a wealth of historical retinal imaging data sourced from eye care service providers such as the Royal Victorian Eye and Ear Hospital and The Australian College of Optometry. Through the collaboration with the Australia Institute of Health and Welfare (AIHW), retinal imaging data can be linked to administrative programme datasets, including the National Death Index (NDI) for death status, Medicare Benefits Schedule (MBS) for Medicare services, and Pharmaceutical Benefits Scheme (PBS) for medication use, as well as hospital and emergency department datasets for important life events like cardiovascular diseases and dementia and ICD diagnosis. So far, more than 500,000 retinal photos and OCT scans from over 100,000 individuals aged 18 years and older have been extracted from one eye care service provider.

Of note is that the data linkage strategy is still in the early stages, and evidence from these projects has yet to be available. The challenges of ensuring data validity and potential selection bias in data linkage projects require careful consideration. Nevertheless, large-scale data linkage projects and AI models could transform the early detection and treatment of various diseases and ultimately benefit patient care and public health strategies.

## Data from biosensors, tele-assessments, and other digital tools

There is substantial interest in the use of home-based and/or wearable biosensors to measure physiologic and kinetic parameters, providing RWD on health outcomes and functional status. Remote assessment of visual function, retinal disease, and optic discs are of high relevance in the field of neuro-ophthalmology and would allow for a greater accessibility to specialised care, facilitating earlier detection, timely intervention and improved management of ocular conditions through telehealth services [35]. Home OCT systems (Notal Vision Home OCT) (NVHO, Notal Vision Inc, SELFF-OCT) validated in age-related macular degeneration [36, 37] and the development of contact lens biosensors that detect real-time changes in intraocular pressure demonstrate this potential [38]. While smartphone and tablet-based devices to assess vision are widespread, these can be single-disease-focused and challenging to access. Nonetheless, emerging technologies such as handheld or mobile phone pupillometry hold promise to neuro-ophthalmologists [39]. The pupillary light reflex and its sensitivity in detecting even mild traumatic brain injury could be translated to other neuro-ophthalmological disease and their monitoring [40, 41].

The potential use of virtual (VR) or augmented reality (AR) for home monitoring of visual fields also holds relevance [40]. VR immerses the user in a virtual environment through use of a specific VR headset, recording the patient’s environment and displaying an image into the visible visual field for assessment. These devices can also track eye movements and detect changes in gaze, improving accuracy and reducing testing time [42]. Safety concerns, given that the patient cannot see their environment, often limit the use of these devices to in-clinic but they are comfortable and can be used by patients in wheelchairs or with other physical constraints. Several devices are already available for glaucoma monitoring (e.g. Easyfield VR (Oculus),Vivid Vision [43], and VirtualEye [44]).

In contrast, Augmented reality (AR), integrates technology with real life. This is commonly used to project images onto an existing image, e.g. virtually trying on sunglasses. The most recent example is the Apple Vision Pro (apple.com/apple-vision-pro). Glasses with AR devices and cameras could map out a scotoma or visual field defect, remap the missing images, and overlay that image onto functioning areas of vision, thus improving a visual field defect [44, 45].

These various tools and technologies hold enormous promise for the future development and expansion of AI algorithms. However, the large quantity of data that can be collected must be integrated with clinical, other imaging, and patient-reported outcomes to understand their role fully. Applications in neuro-ophthalmological conditions are missing but remain of high interest.

## Challenges to RWD collection and standardisation

Despite the promise of RWD in neuro-ophthalmology, several challenges hinder its effective utilisation. One significant hurdle is the heterogeneity and fragmentation of data across different sources. EHRs, for instance, often vary in terms of data structure, coding systems, and quality, making interoperability and data harmonisation challenging. Similarly, disease registries may lack standardised data collection protocols, leading to inconsistencies in data completeness and accuracy. Collaborative research efforts often involve data sharing across jurisdictions, requiring careful navigation of regulatory frameworks and adherence to ethical guidelines to ensure patient confidentiality and data security. Additionally, the sustainability of data sources, particularly disease registries, presents a long-term challenge. The establishment and accuracy of robust data collection systems requires substantial financial investment to maintain infrastructure and guarantee quality. The cost of data curation, including cleaning, validation and integration, adds to the financial burden, as does the investment required for technology, personnel training and regulatory compliance. Advanced analytical techniques such as machine learning or artificial intelligence requires additional computational resources and expertise, with data privacy and security adds further complexity and cost. Obtaining and retaining funding from various sources including government grants, private foundations and industry partnerships plays a crucial role in overcoming these challenges is essential for ensuring viability of data sources long-term.

When generating evidence from RWD, key risks include accounting for known and unknown confounders. Patients who are exposed to therapy, receive a diagnosis, or attend a health service are fundamentally different from those who are not. There are also significant risks of misclassification and ascertainment bias [46]. All proposed analyses of RWD need to consider and plan for this. It is critical that clear and transparent a priori hypotheses are developed before an analysis is conducted to avoid unstructured data mining. Data quality standards should be agreed upon, and this threshold should be explored before an analysis proceeds [47]. Standard methods to control for confounding include ensuring clear restrictions around the included study population, for example, limits to an age group, condition or specific exposure. A comparator group could be created using various propensity score analysis techniques [48].

## Defining core datasets to overcome barriers

RWD and RWE generation in neuro-ophthalmology is in its infancy. The field can benefit from the experience in ophthalmology and neurology to build a meaningful RWD framework. All RWD resources and approaches must consider how to best align to maximise the RWE generated to address critical and unique questions faced in neuro-ophthalmological clinical practice. There is a vital need for stakeholders to develop a conceptual framework for RWD generation in neuro-ophthalmology, starting with the development of a standardised data collection protocol. This is the first step in promoting data quality assurance. While data harmonisation across existing data registries could be considered retrospectively, a prospective approach is ideal. Still, it would involve the establishment of an agreed-upon core dataset or a common data model for each neuro-ophthalmological condition. A priori definitions of measurement procedures and terminology are key (Table 3).

**Table 3:** Definitions [51] and examples of core information requirements in neuro-ophthalmology research.

Term:	Definition:	Relevance to neuro-ophthalmology:
Core dataset.	A collection of variables that serve as the fundamental components across various initiatives and their corresponding minimal datasets. These variables establish foundational data elements across diverse areas of interest and represent critical components of collection.	Variables pertaining to patient demographics (e.g., age, sex) visual function assessments (e.g., visual acuity, visual fields, colour vision), neurological evaluations (e.g., cranial nerve function), ophthalmic imaging metrics (e.g., OCT findings), patient reported outcomes (e.g., quality of life measures).
Minimal dataset.	An agreed upon set of variables within a specific initiative, tailored to address defined objectives or support collaborative efforts. Typically tailored to meet the specific needs of the initiative and may not be universally applicable.	Variables selected for a clinical trial assessing the effectiveness of a novel neuro-ophthalmic intervention (e.g., treatment regimen, adverse events).
Common data element (CDE) [52]	A standardised, precisely defined question that is paired with a set of specific allowable responses, used systematically across different sites, studies or clinical trials to ensure consistent data collection.	Visual acuity evaluation (e.g., “what is the patient’s best corrected visual acuity?”), neurological assessments (e.g., “what is the patient’s cerebrospinal fluid opening pressure?”).

## Discussion

RWD can be collected at scale, allows linkage to various data sources, and has the scope to integrate imaging data and new technology. RWD collection aims to generate RWE that can address knowledge gaps and improve clinical care. The role and use of RWE continue to expand in all fields of medicine. The rare nature of neuro-ophthalmological conditions makes collaboration at scale essential. Although evidence is starting to emerge from disease registries, particularly in IIH, there is still a paucity of RWE in neuro-ophthalmology. This is primarily due to the heterogeneous conditions in neuro-ophthalmology, diverse clinical data being captured, and inconsistencies in primary visual and clinical outcomes. The ability to integrate AI tools within these datasets holds great promise, enabling advanced analytics, pattern recognition, and predictive modelling for a volume of data previously considered too expansive to assess. However, validation of AI tools requires access to large datasets with both disease and disease-free cohorts.

Specific barriers need to be overcome to move RWD forward in neuro-ophthalmology to a place where investigators and clinicians can fully explore the capacity of these data to predict treatment responses, compare the efficacy of treatments for specific conditions and understand long-term outcomes and safety of interventions. As the number of RWD sources grows, it is increasingly critical that the data framework of these data sources is defined in a core data set. This requires input from key stakeholders. Not only could this improve data quality, but also potentially allow for agreement on a basic governance structure and guidance on data-sharing collaborations. It is prudent that we take stock of where we are currently and plan to maximise the potential of RWD resources. Maintaining RWD infrastructure is challenging and expensive and is only sustainable if there is a demonstrable clinical impact.

## Data Availability

Data sharing not applicable to this article as no datasets were generated for this review. All information surrounding registries included can be obtained from the relevant references.
